# Supplementary material for: Pathways of DNA unlinking: A story of stepwise simplification
Source: Sci Rep. 2017 Sep 29;7:12420. doi: 10.1038/s41598-017-12172-2 (PMC5622096; doi:10.1038/s41598-017-12172-2)
Supplement: Supplementary file 1 — Supplementary Methods [file 41598_2017_12172_MOESM1_ESM.pdf]

Pathways of DNA unlinking: A story of stepwise simplification

Robert Stolz[1], Masaaki Yoshida[2,7], Reuben Brasher[3], Michelle Flanner[1], Kai Ishihara[4], David J. Sherratt[5], Koya Shimokawa[2], and Mariel Vazquez*[1,6]

[1]*Department of Microbiology and Molecular Genetics, University of California Davis, Davis, USA*

[2]*Department of Mathematics, Saitama University, Saitama, Japan*

[3]*Microsoft, San Francisco, USA*

[4]*Faculty of Education, Yamaguchi University, Yamaguchi, Japan*

[5]*Department of Biochemistry, University of Oxford, Oxford, UK*

[6]*Department of Mathematics, University of California Davis, Davis, USA*

[7]*Present address: Takasaki City Office, 35-1 Takamatsu-cho, Takasaki, Japan.*

Correspondence and requests for materials should be addressed to M.V. (email: mrlvazquez@ucdavis.edu)

Supplementary Methods

1 Shortest unlinking pathways for the parallel RH 6-cat

In this section we prepare the mathematical results used in the proof of Theorem 2 (main text). A reconnection event is modeled as a system of tangle equations as described in Fig. 1 in the main text. The circular chain represents the starting knot or link, and P is a 2-string tangle that encloses the reconnection sites. Reconnection changes P into R. We assume that each reconnection is modeled as a coherent band surgery, i.e. $P = (0)$ and $R = (w, 0)$ for some integer w. In Shimokawa *et al.*[1] we showed that any minimal pathway to unlink an n-crossing torus link with parallel sites has exactly n steps, and that if each reconnection step is assumed to strictly reduce the complexity of its substrate, then the minimal pathway is unique. In Theorem 2 we assume that reconnection never increases the number of crossings.

Theorem 2. *Consider all shortest unlinking pathways for the parallel RH 6-cat. Assume that the crossing number of any product of local reconnection is smaller or equal to that of its substrate. Then the pathway is one of the 9 shown in Fig. S1.*

1.1 Effect of reconnection on relevant topological invariants

First we review the effect of a reconnection event on certain knot and link invariants, namely the signature $\sigma(L)$, the Jones polynomial, the Q-polynomial and the Arf invariant. The signature $\sigma(L)$ is explained in the Supporting Information of[1] (see also[2]) The definition of the Jones polynomial $V(L;t)$ can be found in a standard knot theory textbook.[2,3] The Jones polynomial of the trefoil knot is $V(3_1;t) = t + t^3 - t^4$. Examples of Jones polynomials of knots can be found, for instance, in *Knotinfo*.[4] Note that their nomenclature of knots is different from ours. Other definitions used are that of the Q-polynomial $Q(L;x)$[5–7] and of Arf invariant.[8]

We consider the case where $P = (0)$ and $R = (w, 0)$ and the orientations of the substrate L and the product L' agree outside P and R, i.e., the case of a coherent band surgery. We assume that L and L' have one or two components. Note that a coherent band surgery always changes the number of components. First we start with a result of Murasugi. We rewrite these results in terms of a local reconnection event, such as site-specific reconnection on circular DNA, or reconnection of knotted vortices in fluids.

Lemma S1. [9] *Let $\sigma(L)$ be the signature of a link L. Consider a local reconnection event represented by a system of tangle equations $N(O+P) = L$ and $N(O+R) = L'$. If $P = (0)$ and $R = (w, 0)$ and the orientations of L and L' agree outside P and R, then $|\sigma(L) - \sigma(L')| \leq 1$.*

The following three results of Kanenobu's relate coherent band surgeries to the Jones polynomial, the Q-polynomial and the Arf invariant.[5,8] Here we restate them in terms of local reconnection.

Lemma S 2. [5] *Let $V(L;t)$ be the Jones polynomial of a link L, and $t = e^{i\pi/3}$. Consider a local reconnection event represented by a system of tangle equations $N(O+P) = L$ and $N(O+R) = L'$. If $P = (0)$ and $R = (w, 0)$ and the orientations of L and L' agree outside P and R, then $V(L;t)/V(L';t) \in \{\pm i, -\sqrt{3}^{\pm 1}\}$.*

Lemma S3. [5] *Let $Q(L;x)$ be the Q-polynomial of a link L, and $\rho(L) = Q(L;(\sqrt{5}-1)/2)$. Consider a local reconnection event represented by a system of tangle equations $N(O+P) = L$ and $N(O+R) = L'$. If $P = (0)$ and $R = (w, 0)$ and the orientations of L and L' agree outside P and R, then $\rho(L)/\rho(L') \in \{\pm 1, \sqrt{5}^{\pm 1}\}$.*

Lemma S4. [8] *Consider a local reconnection event represented by a system of tangle equations $N(O+P) = L$ and $N(O+R) = L'$, where L is a 2-component link with an even linking number. If $P = (0)$ and $R = (w, 0)$ and the orientations of L and L' agree outside P and R, then $Arf(L) = Arf(L')$.*

The following lemma is proved in the Supporting Information of.[1] Here $\chi(L)$ is the Euler characteristic of a link L, which is the largest Euler characteristic of all Seifert surfaces of L.

Lemma S5. [1] *Let L be a non-trivial oriented knot or link and $c(L)$ the crossing number of L. Then the following conclusions hold:*

1. *$\chi(L)$ is at least $2 - c(L)$. Moreover $\chi(L) = 2 - c(L)$ if and only if L is the $(2, c(L))$-torus knot when $c(L)$ is odd or L is the $(2, c(L))$-torus link with parallel orientation when $c(L)$ is even.*

2. *$|\sigma(L)|$ is at most $1 - \chi(L)$.*

3. *$|\sigma(L)|$ is at most $c(L) - 1$. Moreover, $|\sigma(L)| = c(L) - 1$ if and only if L is the $(2, c(L))$-torus knot when $c(L)$ is odd or L is the $(2, c(L))$-torus link with parallel orientation when $c(L)$ is even.*

1.2 Characterization of topologies along the shortest pathways

In this subsection we prove Theorem 2. From Lemma S5, the absolute value of the signature of a knot or link with at most 6-crossings is at most 5. First we characterize the signature of knots and links

along any shortest unlinking pathway.

Lemma S6. *Suppose an unlinking pathway from the RH 6-cat (signature* $= -5$*) is shortest. Then the signature of the first product (resp. 2nd, 3rd, 4th, 5th and last) is* -4 *(resp.* -3, -2, -1, 0, 0*).*

Proof. From Lemma S1 we know that a local reconnection event changes the signature by at most one. Also, the signature of a knot is an even integer.[9] □

Next we consider split links.

Lemma S7. *No split link other than the unlink appears in a shortest unlinking pathway.*

Proof. Let L be a 2-component split link consisting of knots K_1 and K_2. We know that $\sigma(L) = \sigma(K_1)+\sigma(K_2)$, and $\sigma(K_1)$ and $\sigma(K_2)$ are even integers.[9] Then $\sigma(L)$ is an even integer. However, from Lemma S6 every link other than the unlink in a shortest path has an odd integral signature. Hence a 2-component split link L cannot appear in a shortest unlinking pathway unless $L = 0_1^2$. □

Based on Lemma S7, we consider non-split links. Next, we characterize knots and 2-component links with up to six crossings and non-positive signatures.

Lemma S8. *Let* L *be a 2-component link with crossing number at most 6 and let* K *be a knot with crossing number at most 6.*

1. $\sigma(L) = -5$ *if and only if* $L = 6_1^2$.
2. $\sigma(K) = -4$ *if and only if* $K = 5_1$ *or* $3_1\#3_1$.
3. $\sigma(L) = -3$ *if and only if* $L = 4_1^2$, 6_2^2, 6_3^2 *or* $3_1\#2_1^2$.
4. $\sigma(K) = -2$ *if and only if* $K = 3_1$, 5_2 *or* 6_2.
5. $\sigma(L) = -1$ *if and only if* $L = 2_1^2$, $4_1^{2*\prime}$, 5_1^2, $6_1^{2*\prime}$, $6_3^{2*\prime}$ *or* $3_1\#2_1^{2\prime}$.
6. $\sigma(K) = 0$ *if and only if* $K = 0_1$, 4_1, 6_1, 6_1^*, 6_3 *or* $3_1\#3_1^*$.

1.3 Characterization of shortest unlinking pathways

The following Propositions S9-S17 are used to prove Theorem 2.

Proposition S 9 (Step I)**.** *Consider a local reconnection event represented by a system of tangle equations* $N(O+P)=6_1^2$ *and* $N(O+R)=K$*, where* K *is a knot with crossing number at most 6. If* $P=(0)$ *and* $R=(w,0)$ *and the orientations of* 6_1^2 *and* K *agree outside* P *and* R*, then* $K=5_1$ *or* $3_1\#3_1$*. Moreover solutions to the tangle equations exist for those cases.*

Proof. By Lemma S5, $\sigma(K)=-4$. Since the crossing number of K is at most 6, by Lemma S8, $K=5_1$ or $3_1\#3_1$. The corresponding system of tangle equations can be solved. Two solutions are illustrated in Fig. S2 (A). □

In the second step, first we consider links obtained by a local reconnection event on 5_1.

Proposition S10 (Step IIa)**.** *Consider a local reconnection event represented by a system of tangle equations* $N(O+P)=5_1$ *and* $N(O+R)=L$*, where* L *is a 2-component link with crossing number at most 5 and* $\sigma(L)=-3$*. If* $P=(0)$ *and* $R=(w,0)$ *and the orientations of* 5_1 *and* L *agree outside* P *and* R*, then* $L=4_1^2$ *or* $3_1\#2_1^2$*. Moreover solutions to the tangle equations exist for those cases.*

Proof. From $\sigma(L)=-3$ and $c(L)\leq 5$, by Lemma 8, $L=4_1^2$ or $3_1\#2_1^2$. The corresponding system of tangle equations can be solved. Two solutions are illustrated in Fig. S2(B). □

We now consider links obtained by a local reconnection event on $3_1\#3_1$.

Proposition S11 (Step IIb)**.** *Consider a local reconnection event represented by a system of tangle equations* $N(O+P)=3_1\#3_1$ *and* $N(O+R)=L$*, where* L *is a 2-component link with crossing number at most 6 and* $\sigma(L)=-3$*. If* $P=(0)$ *and* $R=(w,0)$ *and the orientations of* $3_1\#3_1$ *and* L *agree outside* P *and* R*, then* $L=6_3^2$ *or* $3_1\#2_1^2$ *Moreover solutions to the tangle equations exist for those cases.*

Proof. By $\sigma(L)=-3$ and $c(L)\leq 6$, we have $L=4_1^2$, 6_2^2, 6_3^2 or $3_1\#2_1^2$. Since $V(4_1^2;\omega)=V(6_2^2;\omega)=-i$ and $V(3_1\#3_1;\omega)=-3$, 4_1^2 and 6_2^2 do not satisfy Lemma 2. The corresponding system of tangle equations can be solved. Two solutions are illustrated in Fig. S2(C). □

In the third step first consider knots obtained by a local reconnection event on the link 6_3^2.

Proposition S12 (Step IIIa)**.** *Consider a local reconnection event represented by a system of tangle equations* $N(O+P)=6_3^2$ *and* $N(O+R)=K$*, where* K *is a knot with crossing number at most 6 and* $\sigma(K)=-2$*. If* $P=(0)$ *and* $R=(w,0)$ *and the orientations of* 6_3^2 *and* K *agree outside* P *and* R*, then* $K=5_2$*. Moreover solutions to the tangle equations exist for this case.*

Proof. If $\sigma(K) = -2$ and $c(K) \leq 6$, we have $K = 5_2$, 3_1 or 6_2. Since $V(6^2_3;\omega) = \sqrt{3}$ and $V(6_2;\omega) = 1$, 6_2 does not satisfy Lemma 2. Since $lk(6^2_3) = 2$, $\mathrm{Arf}(6^2_3) = 0$ and $\mathrm{Arf}(3_1) = 1$, 3_1 does not satisfy Lemma 4. The corresponding system of tangle equations can be solved. The solution is illustrated in Fig. S2(D). □

Proposition S13 (Step IIIb)**.** *Consider a local reconnection event represented by a system of tangle equations $N(O + P) = 3_1\#2^2_1$ and $N(O + R) = K$, where K is a knot with crossing number at most 5 and $\sigma(K) = -2$. If $P = (0)$ and $R = (w, 0)$ and the orientations of $3_1\#2^2_1$ and K agree outside P and R, then $K = 5_2$ or 3_1. Moreover solutions to the tangle equations exist for these cases.*

Proof. If $\sigma(K) = -2$ and $c(K) \leq 5$, we have $K = 5_2$, 3_1. The corresponding system of tangle equations can be solved. Two solutions are illustrated in Fig. S2(E). □

Proposition S14 (Step IIIc)**.** *Consider a local reconnection event represented by a system of tangle equations $N(O + P) = 4^2_1$ and $N(O + R) = K$, where K is a knot with crossing number at most 4 and $\sigma(K) = -2$. If $P = (0)$ and $R = (w, 0)$ and the orientations of 4^2_1 and K agree outside P and R, then $K = 3_1$. Moreover solutions to the tangle equations exist for this case.*

Proof. If $\sigma(K) = -2$ and $c(K) \leq 4$, we have 3_1. The corresponding system of tangle equations can be solved. The solution is illustrated in Fig. S2(F). □

Next we consider the 4th step. First we consider links obtained by a local reconnection on 5_2.

Proposition S15 (Step IVa)**.** *Consider a local reconnection event represented by a system of tangle equations $N(O + P) = 5_2$ and $N(O + R) = L$, where L is a 2-component link with crossing number at most 5 and $\sigma(L) = -1$. If $P = (0)$ and $R = (w, 0)$ and the orientations of 5_2 and L agree outside P and R, then $L = 2^2_1$ or $4^{2*'}_1$. Moreover solutions to the tangle equations exist for these cases.*

Proof. By $c(L) \leq 5$ and $\sigma(L) = -1$, $L = 2^2_1$, $4^{2*'}_1$, 5^2_1 or $3_1\#2^{2'}_1$. Since $V(5_2;\omega) = -1$ and $V(3_1!\#2^{2'}_1) = -\sqrt{3}$, by Lemma 2, $3_1\#2^{2'}_1$ can not be the product of the local reconnection. Since $\mathrm{Arf}(5_2) = 0$, $lk(5^2_1) = 0$, $\mathrm{Arf}(5^2_1) = 1$, there is no band surgery from 5_2 to 5^2_1 by lemma S4. Moreover solutions to the tangle equations exist for the case where $L = 2^2_1$ and $4^{2*'}_1$ as in Fig. S2(G). □

Proposition S16 (Step IVb)**.** *Consider a local reconnection event represented by a system of tangle equations $N(O + P) = 3_1$ and $N(O + R) = L$, where L is a 2-component link with crossing number at most 3 and $\sigma(L) = -1$. If $P = (0)$ and $R = (w, 0)$ and the orientations of 3_1 and L agree outside*

P and R, then $L = 2^2_1$. The corresponding system of tangle equations can be solved. The solution is illustrated in Fig. S2(H).

Proof. By $c(L) \leq 3$ and $\sigma(L) = -1$, $L = 2^2_1$. Moreover solutions to the tangle equations exist for this case as in Fig. S2(H). □

Proposition S17 (Step V)**.** *Consider a local reconnection event represented by a system of tangle equations $N(O+P) = 4^{2*\prime}_1$ and $N(O+R) = K$, where K is a knot with crossing number at most 4 and $\sigma(K) = 0$. If $P = (0)$ and $R = (w, 0)$ and the orientations of $4^{2*\prime}_1$ and K agree outside P and R, then $K = 0_1$. Moreover solutions to the tangle equations exist for this case.*

Proof. Since K is a knot with at most 4 crossings and $\sigma(K) = 0$, we have $K = 0_1$, or 4_1.

By $\rho(4^{2*\prime}_1) = 1$ and $\rho(4_1) = -\sqrt{5}$, by Lemma S3, 4_1 can not be obtained. In fact, there is a coherent band surgery from $4^{2*\prime}_1$ to 0_1. □

Suppose the substrate is 2^2_1. Then the possible product satisfying the assumption is 0_1. There is a solution of the tangle equation $N(O+P) = 2^2_1$ and $N(O+R) = 0_1$ as in Fig. S2(I). But a band surgery is unique up to isotopy.[10,11]

1.4 Characterization of mechanisms

We here add details to the statement of Proposition 3 in the main text to exhibit solutions that are relevant to any local reconnection event.

Proposition 3. *A.*[12] *Suppose $N(O+P) = 5_2$, $N(O+R) = 2^2_1$, $P = (0)$ and $R = (w, 0)$. Then $O = (\frac{7}{-7w-2})$. In particular,*

1. *if $P = (0)$ and $R = (-1)$, then $O = (\frac{7}{5})$.*
2. *if $P = (0)$ and $R = (0, 0)$, then $O = (-\frac{7}{2})$.*
3. *if $P = (0)$ and $R = (1)$, then $O = (-\frac{7}{9})$. (See Fig. S3(A).)*

B.[12] *Suppose $N(O+P) = 5_2$, $N(O+R) = 4^{2*\prime}_1$, $P = (0)$ and $R = (w, 0)$. Then $O = (\frac{7}{-7w-4})$. In particular,*

1. *if $(P, R) = ((0), (-1))$, then $O = (\frac{7}{3})$.*

2. if $(P,R) = (0),(0,0)$, *then* $O = (-\frac{7}{4})$.

3. if $(P,R) = ((0),(1))$, *then* $O = (-\frac{7}{11})$. *(See Fig. S3(B).)*

C.[13] *Suppose* $N(O+P) = 4_1^{2*'}$, $N(O+R) = 0_1$, $P = (0)$ *and* $R = (w,0)$. *Then* $O = (\frac{4}{-4w-1})$. *In particular,*

1. if $P = (0)$ *and* $R = (-1)$, *then* $O = (\frac{4}{3})$.

2. if $P = (0)$ *and* $R = (0,0)$, *then* $O = (-4)$.

3. if $P = (0)$ *and* $R = (1)$, *then* $O = (-\frac{4}{5})$. *(See Fig. S3(C).)*

First we cite a theorem which characterizes the solutions of a system of tangle equations where the substrate is a genus one rational knot $N(\frac{4mn-1}{2m})$, and the product is an anti-parallel $2k$-cat $N(2k)$.[12] Here $N(\frac{a}{b})$ indicates the knot or link obtained by applying the numerator operation on the rational tangle classified by the rational number $\frac{a}{b}$.

Theorem S18. *[Theorem 3.1*[12]*] Suppose*

$$(*)\ N(O+(0)) = N\left(\frac{4mn-1}{2m}\right) \quad and \quad N(O+\left(\frac{1}{w}\right)) = N(2k)$$

and the rational tangle surgery corresponds to a coherent band surgery. If $(2,2k)$*-torus link as linking number* k *where* $|k| > 2$, *then* $(*)$ *has no solution. If the* $(2,2k)$*-torus link has linking number* $-k$, *then one of the following holds.*

1. $k = m$ *and* $O = \left(\frac{4mn-1}{-w(4mn-1)+2m}\right)$

2. $k = n$ *and* $O = \left(\frac{4mn-1}{-w(4mn-1)+2n}\right)$

3. $k = m+n+1$ *and* $O = \left(\frac{1}{2m+1} + \frac{-1}{2n+1}\right) \circ (1,-(w+1),0)$ *or* $O = \left(\frac{1}{2n+1} + \frac{-1}{2m+1}\right) \circ (1,-(w+1),0)$.

4. $k = m+n-1$ *and* $O = \left(\frac{1}{2m-1} + \frac{-1}{2n-1}\right) \circ (-1,-(w-1),0)$ *or* $O = \left(\frac{1}{2n-1} + \frac{-1}{2m-1}\right) \circ (-1,-(w-1),0)$.

Proof of Proposition 3A. Since $N(O+P) = 5_2$ and $N(O+R) = 2_1^2$, from Theorem S18 we have $k = m = -1$ and $n = -2$ or $k = n = -1$ and $m = -2$. Then $O = \left(\frac{7}{-7w-2}\right)$. If $w = -1$, $R = (-1)$ and $O = \left(\frac{7}{5}\right)$. If $w = 0$, $R = (0,0)$ and $O = \left(-\frac{7}{2}\right)$. If $w = 0$, $R = (1)$ and $O = \left(-\frac{7}{9}\right)$. □

Proof of Proposition 3B. Since $N(O+P) = 5_2$ and $N(O+R) = 4_1^{2*'}$, from Theorem S18 we have

$k = m = n = -2$. Then $O = \left(\frac{7}{-7w-4}\right)$. If $w = -1$, $R = (-1)$ and $O = \left(\frac{7}{3}\right)$. If $w = 0$, $R = (0, 0)$ and $O = \left(-\frac{7}{4}\right)$. If $w = 0$, $R = (1)$ and $O = \left(-\frac{7}{11}\right)$. □

The mechanism of the case where the substrate is $4_1^{2*\prime}$ (anti-parallel 4-cat) and the product is 0_1 can be characterized.[13] First we cite a result of Ernst and Sumners.

Lemma S19. [14] *If X and A are two rational tangles, with classifying rational numbers $\frac{u}{v}$ and $\frac{x}{y}$, respectively, then $N(X + A) = b(\alpha, \beta)$ is a 4-plat where $\alpha = |xv + yu|$, and β is determined as follows:*

1. *If $\alpha = 0$ then $\beta = 1$;*
2. *If $\alpha = 1$ then $\beta = 1$;*
3. *If $\alpha > 1$, then β is determined uniquely by $0 < \beta < \alpha$ and $\beta \equiv \sigma(vy' + ux')(mod\ \alpha)$, where $\sigma = sign(vx + yu)$ and y' and x' are integers such that $xx' - yy' = 1$.*

Proof of Proposition 3C. By Corollay 1.5 in Hirasawa and Shimokawa,[11] O is a rational tangle. Let $O = (\frac{u}{v})$. Since $P = (0)$ and $N(O + P) = 4_1^{2*\prime} = b(4, 3)$, by Lemma S19, $4 = |0 + u| = |u|$. Since $R = (\frac{1}{w})$ and $N(O+R) = 0_1 = b(1, 0)$, by Lemma S19, $1 = |v+uw|$. Then we have $O = (\frac{u}{v}) = (\frac{4}{-4w+1})$ or $(\frac{4}{-4w-1})$. From the conclusion 3 of Lemma S19, we can conclude that $O = (\frac{4}{-4w-1})$ □

2 Supplementary Numerical Methods

2.1 Generation of lattice substrate with BFACF as a Multiple Markov process

A variety of Monte Carlo methods have been used to model circular DNA in solution.[15,16] Of particular interest is the Monte Carlo based BFACF algorithm which samples uniformly the space of self-avoiding lattice polygons (SAPs) of an arbitrary topological type.[17] While lattice models have been criticized for not capturing physical features of DNA as a wormlike chain model would, they present with multiple theoretical and computational advantages over methods in $\mathbb{R}^3$. The BFACF algorithm is of particular interest to our study because it randomly modifies SAPs without changing the topology of the conformation, it is ergodic within the state space of SAPs of a given knot type, and is computationally efficient (our computer implementation runs in constant time on the number of edges). The ergodicity of the algorithm within each given topology implies that long enough runs on a closed SAP of a knot/link type L will yield a uniform sample of all chains of type L.

The BFACF algorithm acts on the edges of a SAP by performing one of three possible moves illustrated in Fig. S4. After choosing an edge at random, one of the moves and a direction can be selected randomly with equal probability. The choice of move and direction can be optimized by using the conformation of edges adjacent to the selected edge to rule out moves that would violate self-avoidance. The probability of selecting any of the remaining moves are re-weighted to preserve the uniformity of selection while rejecting far fewer BFACF moves. The probability of acceptance associated to each move is a function of the variables z, q, and n, where n is the current length of the polygon and z and q are fugacity parameters. The algorithm computes the acceptance probability of the selected move and compares it to a uniform random number generated on the interval $[0, 1]$. If the move is accepted but would violate the self-avoiding property of the SAP, it is rejected. If the move is rejected, BFACF makes no changes to the selected edge and the process is repeated with the selection of a new edge at random. Depending on the choice of z, and q, equations 1, 2, and 3 yield different acceptance probabilities for the three moves. Higher z values result in a higher probability of accepting +2 moves, which over many BFACF moves results in conformations that are on average longer. In other words, each z value has a different average length associated with it for any given topology. The parameter q is typically fixed to a positive integer value ≥ 1. For a fixed z, increasing q results in a higher probability of accepting the +2 move at shorter polygon lengths. This increase in the acceptance probability of the +2 move scales back as the polygon increases in length. The ultimate effect is that in the most common scenarios, $q = 2$ and $q = 3$, the average length and length variance of the sampled conformations increases significantly. Certain topologies, such as the unknot which tends to decrease to its minimal length of 4, require $q \geq 3$ to achieve an average length significantly above 20. For further information on the q parameter the reader is referred to the work of Szafron.[18,19]

$$P(+2) = \frac{(n+2)^{q-1}z^2}{n^{q-1} + 3(n+2)^{q-1}z^2} \tag{1}$$

$$P(-2) = \frac{n^{q-1}}{n^{q-1} + 3(n+2)^{q-1}z^2} \tag{2}$$

$$P(0) = \frac{P(+2) + P(-2)}{2} \tag{3}$$

Note that if we take $q = 1$, the BFACF move acceptance probabilities no longer depend on the current length of the polygon and these equations simplify to the following:[17]

$$P(+2) = \frac{z^2}{1 + 3z^2} \tag{4}$$

$$P(-2) = \frac{1}{1+3z^2} \tag{5}$$

$$P(0) = \frac{1+z^2}{2(1+3z^2)} \tag{6}$$

2.2 CMC BFACF

As was mentioned in the previous section, larger values of the parameters z and q produce higher average length, length variance, and dependence between consecutive samples with a fixed number of BFACF iterations between them. Because BFACF changes at most three edges per move regardless of the overall length of the conformation, more BFACF iterations are needed between essentially independent samples drawn from distributions with longer average lengths. For the purpose of modeling DNA, we frequently need to sample knots and links at lengths that require many steps between samples. For biologically important topologies such as the unknot, the number of steps between samples must be prohibitively large. To overcome this problem we use the Composite Markov Chain (CMC) Monte Carlo approach proposed by Orlandini[20] for the Pivot algorithm and later extended to BFACF and θ-BFACF by Szafron.[18,19]

CMC BFACF works by running a number of simulations on the same topology in parallel, spread out across a range of z parameters. After a chosen fixed number of BFACF iterations in all simulations, in our case 5, we choose two simulations with adjacent z values uniformly at random and attempt to swap the conformations. For 'adjacent' simulations i and j, we will accept a swap with probability:

$$\min\left(1, \frac{\pi(Z_i)}{\pi(Z_j)}^{\pi(Z_j)-\pi(Z_i)}\right) \tag{7}$$

where $\pi(Z_i)$ is the current length of the conformation in simulation i with fugacity parameter Z and $Z_i > Z_j$. After the swap attempt, the simulations continue to the next swap attempt.

In order to maximize the increased speed of conformation randomization, it is desirable to ensure that all the simulations are swapping with each other at an approximately equivalent rate. Using work by Szafron as a precedent,[18,19] we choose that rate to be 0.8. To ensure consistent swap rate between simulations, we perform a calibration phase of 1×10^7 BFACF iterations before sampling. While calibrating, we attempt swaps every 5 BFACF iterations and track the swap acceptance rate. For any adjacent simulations i and j for which the swap acceptance rate falls below 0.8, we add a new

simulation with a fugacity parameter equal to:

$$e^{\frac{ln(Z_j)-ln(Z_i)}{2}} \tag{8}$$

2.3 Batch-mean Analysis

CMC BFACF provides a way to sample conformations from slowly converging distributions more efficiently than the original BFACF algorithm. However, without more specialized techniques, we still require sufficient BFACF steps between samples and a method of estimation to claim that they are essentially independent from each other. To evade this ultimately very computationally expensive requirement, we employ Batch-Mean Analysis (BMA).[21] Intuitively, BMA corrects for dependence by averaging consecutive samples into "blocks" which are tested for independence from other blocks. BMA provides a way to sample at a frequent fixed interval and correct for dependent samples in later analysis of the results without discarding any dependent samples. Each round of sampling occurs after $20,000$ BFACF moves in each chain. Conformation length and reconnection site data is recorded directly to a file and is used to determine the independent block size and in subsequent statistical analysis. In order to save storage space and minimize read and write time, the conformations containing reconnection sites are saved to a file are stored in a binary representation of the NEWSUD format.[22]

2.4 Ratio-estimation

Since we can not sample directly from the population of conformations within each topology that have at least one site meeting our strict reconnection criteria, a simple proportion that estimates the probability of transitioning from one topology to another will be biased. Another perspective is that in the proportions we want to estimate, both the numerator and denominator are random variables, and we can not sample from either population directly. To correct for bias introduced by this setting, we apply ratio estimation corrections.[21]

2.5 Recombo helper

Our exploration of transition pathways has added many new topologies to our substrate list over the course of this study. Each new topology adds computational complexity to the simulations, eventually requiring high levels of automation, and specialized support software to generate scripts, analyze

results and produce visualizations. *Recombo* Helper is a python based utility suite developed to meet these needs. Its first feature is the ability to automatically generate simulation and conformation identification scripts for an arbitrary list of topologies for use with Linux based computing clusters. We used this capability to generate the scripts that in turn generated the data for this study. It also combines the identification of each conformation after a reconnection event with the length and reconnection site data recorded during sampling to produce a sequence of independent block means and subsequent ratio-estimation confidence intervals. *Recombo* Helper organizes the results into matrices and incorporates automatic error checking by searching for theoretically impossible transition that appear in the data. After all the results have been analyzed, it also has the ability to generate karyotype and transition files needed by the Circos program to produce visualizations of the transition network.[23] The Supplementary Data provided online is a spreadsheet reporting on the transitions probabilities (presented as percentages) from our *Recombo* experiments.

3 Knot and Link nomenclature and knot identification

Note that the nomenclature used here departs significantly from that in some of the band surgery literature[24–26] which is based on the nomenclature proposed by Kanenobu.[5,8] For example 3_1 in this paper corresponds to $3_1!$ in Shimokawa *et al.*[24] In the case of links 4^2_1 here corresponds to $4^2_1!$ in Ishihara and Shimokawa[24] and $4^{2\prime}_1$ here corresponds to $4^{2\prime}_1!$ in Ishihara and Shimokawa.[24] Likewise our writhe-guided nomenclature deviates from that in Knotplot.[27] For example, in the Knotplot nomenclature L_a indicates the 2-component link of type L with the chirality and site orientation that appears in the Knotplot link zoo, L_b is obtained from L_a by reversing the orientation of one of the components, L_c is the mirror image of L_b, and L_d is the mirror image of L_a. 4^2_1 here corresponds to 4^2_{1b} in Knotplot. The writhe-guided nomenclature for all prime knots with up to 10 crossings as proposed in Brasher *et al.*[28] can be obtained from the authors upon request. Fig. S5 summarizes the nomenclature used for a representative set of two component links, and how it compares to that in several sources.[5,8,27,29] These figures allow the reader to unambiguously determine the identity of a knot or link of interest for small crossing numbers. For 2-component links with higher crossing number we follow the Knotplot nomenclature.[27]

Knot identification: We use HOMFLY-PT in order to identify the topologies of knots and links obtained after reconnection.[30,31] HOMFLY-PT is a topological invariant, meaning that if two knotted conformations K_1 and K_2 have different HOMFLY-PT polynomials, then the knot type of K_1 is nec-

essarily different to the knot type of K_2. For more information about polynomial invariants of knots the reader is referred to a standard knot theory textbook.[3,32] In general the HOMFLY-PT polynomial distinguishes a chiral knot from its mirror images and it behaves as a perfect invariant for prime knots with 7 or fewer crossings. There are however some ambiguities in the calculation of HOMFLY-PT for more complex knots and links. For example, chiral knots and links in the following set have the same HOMFLY-PT as their mirror images: $\{8_{17}, 9_{42}, 10_{48}, 10_{71}, 10_{91}, 10_{104}, 10_{125}\}$. Also, the following pairs share the same HOMFLY-PT: $\{(5_1, 10_{132}); (8_8, 10_{129}); (8_{16}, 10_{156}); (9_{12}, 4_1\#5_2); (10_{25}, 10_{56}); (10_{40}, 10_{103}); (9^2_{12}, 9^2_{34})\}$. In the case of oriented two component links, there are several pairs with the same HOMFLY-PT, which correspond to links of the same type, but with different orientation on the components.

4 Data

Fig. is a Circos figure illustrating observed transitions for 271 topologies within nullification distance 3 of the 9 unique minimal pathways, with 726 topologies being analyzed in total, including those in non-minimal pathways. This figure is a visual representation of this subset of the transition probability matrix obtained from the computer simulations here presented. The full dataset is included as a spreadsheet in the Supplementary Data file online (StolzSupplementaryData.xls).

Supplementary figures and captions

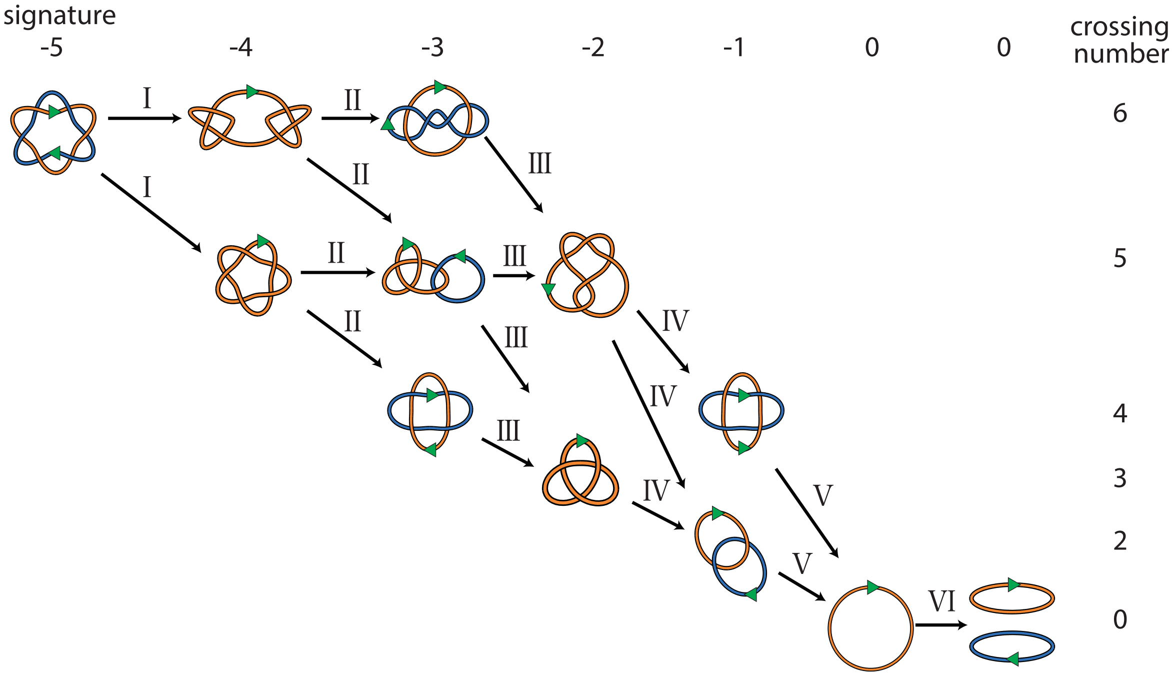

Figure S1: All shortest pathways taking the link 6^2_1 to the unlink and satisfying the assumption that each product along the pathway is a knot or a 2-component link and that no reconnection event increases the number of crossings of its substrate. The Roman numerals over each transition correspond to the steps of the proof indicated in Propositions S9-S17.

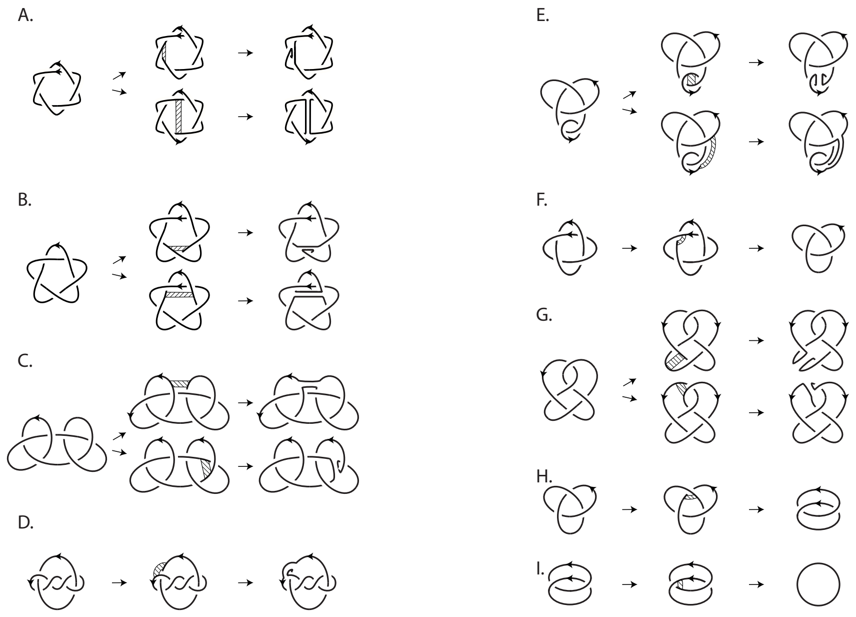

Figure S2: **A.** From Proposition S9, if the substrate is 6_1^2 then the product is either 5_1 or $3_1\#3_1$. Solutions to the tangle equations, which are shown as band surgeries, exist in both cases. **B.** From Proposition S10, if the substrate is 5_1 then the product is either 4_1^2 or $3_1\#2_1^2$. Solutions to the tangle equation, which are shown as band surgeries, exist in both cases. **C.** From Proposition S11, if the substrate is $3_1\#3_1$ then the product is either 6_3^2 or $3_1\#2_1^2$. Solutions to tangle equations, which are shown as band surgeries, exist in both cases. **D.** From Proposition S12, if the substrate is 6_3^2 then the product is 5_2. A solution to the tangle equation, which is shown as a band surgery, exists in this case. **E.** From Proposition S13, if the substrate is $3_1\#2_1^2$ then the product is either $K = 5_2$ or 3_1. Solutions to tangle equations, which are shown as band surgeries, exist in both cases. **F.** From Proposition S14, if the substrate is 4_1^2 then the product is 3_1. A solution to the tangle equation, which is shown as a band surgery, exists in this case. **G.** Band surgeries from 5_2 to 2_1^2 and $4_1^{2*\prime}$. We know that band surgeries are unique up to isotopy for these cases.[12] **H.** From Proposition S15, if the substrate is 3_1 then the product is 2_1^2. A solution to the tangle equation, which is shown as a band surgery, exists in this case. **I.** If the substrate is 2_1^2 then the product is 0_1. A solution to the tangle equation, which is shown as a band surgery, exists and unique up to isotopy.

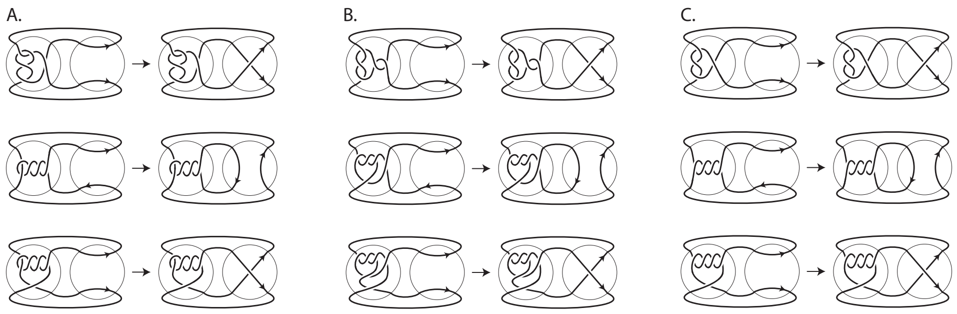

Figure S3: **A.** Three solutions of tangle equations $N(O+P) = 5_2$ and $N(O+R) = 2^2_1$. Here $P = (0)$ and $R = (w, 0)$, where $w = -1, 0$ or 1. **B.** Three solutions of tangle equations $N(O+P) = 5_2$ and $N(O+R) = 4_1^{2*\prime}$. Here $P = (0)$ and $R = (w, 0)$, where $w = -1, 0$ or 1. **C.** Three solutions of tangle equations $N(O+P) = 4_1^{2*\prime}$ and $N(O+R) = 0_1$. Here $P = (0)$ and $R = (w, 0)$, where $w = -1, 0$ or 1.

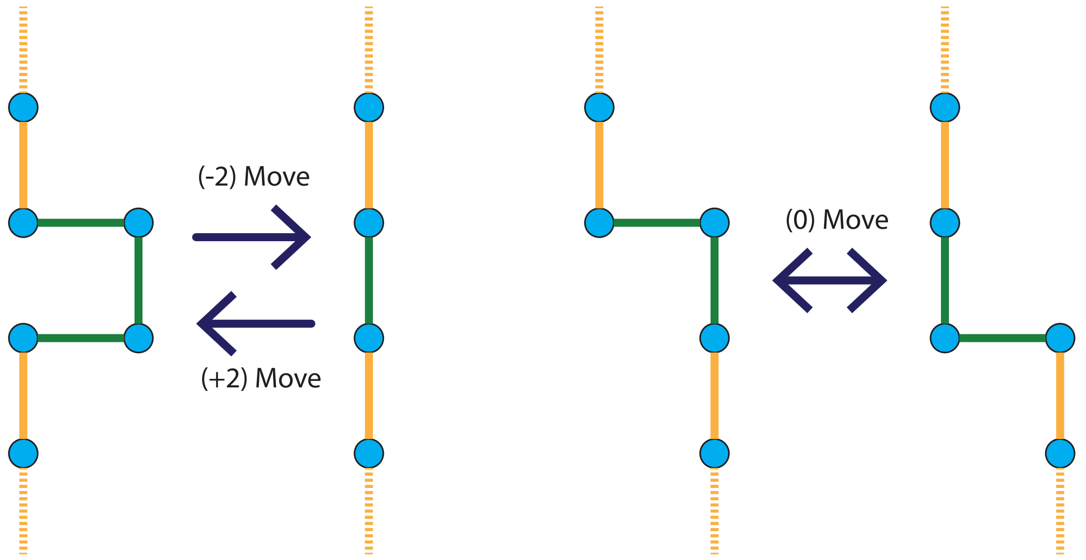

Figure S4: The figure illustrates the three BFACF moves: $+2$ move, -2 move and 0 move. The $+2$ move takes any edge segment, and pushes it out in a random direction to increase the length of the polygon at that location by two. The -2 move is the reverse of the $+2$ move and reduces the length of the polygon by 2. The 0 move leaves the length of the polygon fixed while changing a vertex and its attached edges.

Diagram	Writhe	KnotPlot	Kanenobu	Hoste
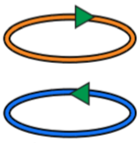	0_1^2	0.2.1.a	0_1^2	$0_1^2{++}$
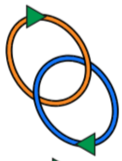	2_1^2	2.2.1.a	$2_1^2!$	$2_1^2{++}$
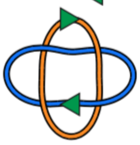	4_1^2	4.2.1.b	$4_1^2!$	4_1^2*+-
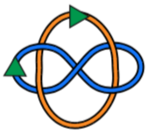	5_1^2	5.2.1.a	5_1^2	5_1^2*++
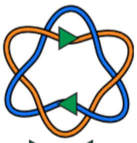	6_1^2	6.2.1.a	$6_1^2!$	$6_1^2{++}$
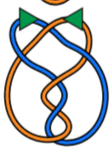	6_2^2	6.2.2.a	$6_2^2!$	$6_2^2{++}$
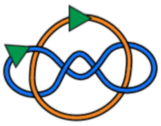	6_3^2	6.2.3.b	$6_3^2!$	$6_3^2{++}$
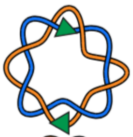	8_1^2	8.2.1.b	$8_1^2!$	$8_1^2{++}$
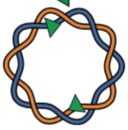	10_1^2	10.2.1.d	$10_1^2!$	$10_1^2{++}$

Figure S5: Link Table with nomenclature comparisons to other known tables.[5,8,27,29]

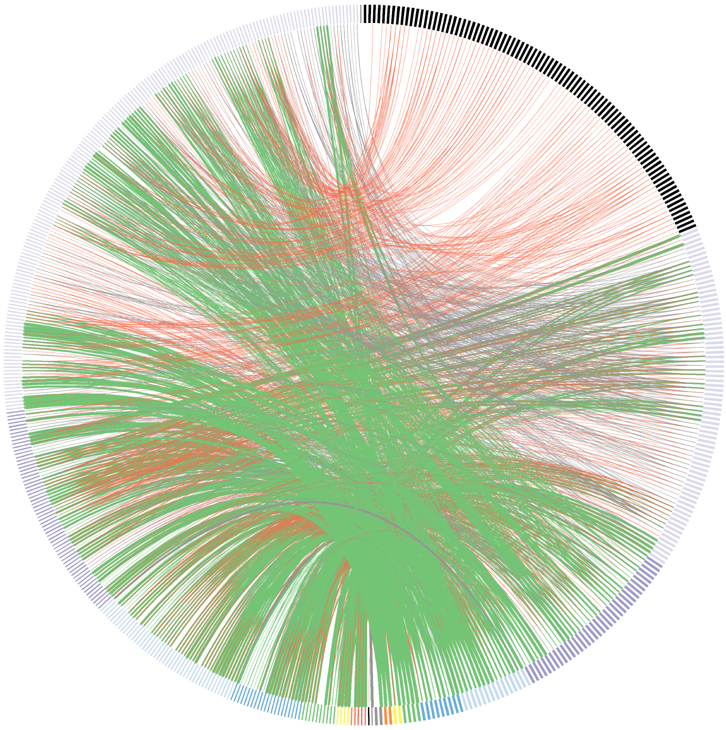

Figure S6: Circos figure indicating the reconnection transitions between all topologies that are members of at least one minimal pathway from the 9_1 knot. Each dash along the circumference corresponds to a topology. The dashes are colored by the minimal crossing number of the corresponding topology, with colors ranging from violet to blue as minimal crossing number decreases from 9 to 1. 10 crossing knots and the unlink are shown as black to emphasize that they were not used as substrate topologies, and therefore have no outgoing transitions. Knots are arranged on the right hemisphere, and two component links on the left. An arc between two topologies K and L indicates a number of reconnection events taking K into L or vice versa have been observed in our simulations. The thickness of the arcs corresponds to the directed transition probability between two topologies. Transitions with an observed probability < 0.2 are thickened to be more visible. Transitions are colored the change in minimal crossing number they result in, with transitions that decrease, increase, and maintain minimal crossing number colored green, red, and grey respectively. If no arc appears between pair (K, L), this means that no reconnection event took K into L or vice versa.

Supplementary Data: The spreadsheet StolzSupplementaryData.xlsx contains point estimates for the transition probabilities between any pair of knot/link topologies in the computational study. The second sheet contains Ratio-estimation confidence intervals at significance level $\alpha = 0.05$, presented

as lower-bound | upper-bound. The substrate topologies are arranged by increasing minimal crossing number in each row. There are 491 substrate topologies, including almost all knots and links with 9 or fewer crossings and their symmetry types. The columns indicate the observed destination topologies. There are 881 substrate and product topologies, including prime and composite knots and links with up to 10 crossings, as well as some split knots or links.

References

[1] Shimokawa, K., Ishihara, K., Grainge, I., Sherratt, D. J. & Vazquez, M. FtsK-dependent XerCD-*dif* recombination unlinks replication catenanes in a stepwise manner. *Proc. Natl. Acad. Sci. USA* **110**, 20906–20911 (2013). arXiv:http://www.pnas.org/content/110/52/20906.full.pdf+html.

[2] Murasugi, K. *Knot theory and its applications* (Birkhäuser Boston Inc., Boston, MA, 2008).

[3] Adams, C. *The Knot Book: An Elementary Introduction to the Mathematical Theory of Knots* (AMS Chelsea, Providence, RI, 2001).

[4] Cha, J. C. & Livingston, C. Knotinfo: Table of knot invariants, http://www.indiana.edu/~knotinfo (2016).

[5] Kanenobu, T. Band surgery on knots and links. *J. Knot Theory Ramifications* **19**, 1535–1547 (2010).

[6] Brandt, R. D., Lickorish, W. B. R. & Millett, K. A polynomial invariant for unoriented knots and links. *Inv. Math.* **84**, 563–573 (1986).

[7] Ho, C. F. A polynomial invariant for knots and links–preliminary report. *Abstracts Amer. Math. Soc.* **6**, 300 (1985).

[8] Kanenobu, T. Band surgery on knots and links, II. *J. Knot Theory Ramifications* **21**, 1250086–108 (2012).

[9] Murasugi, K. On a certain numerical invariant of link types. *Trans. Amer. Math. Soc.* **117**, 387–422 (1965).

[10] Thompson, A. Knots with unknotting number one are determined by their complements. *Topology* **28**, 225–230 (1989).

[11] Hirasawa, M. & Shimokawa, K. Dehn surgeries on strongly invertible knots which yield lens spaces. *Proc. Amer. Math. Soc.* **128**, 3445–3451 (2000).

[12] Darcy, I. K., Ishihara, K., Medikonduri, R. K. & Shimokawa, K. Rational tangle surgery and Xer recombination on catenanes. *Algebr. Geom. Topol.* **12**, 1183–1210 (2012). arXiv:`https://arxiv.org/abs/1108.0724`.

[13] Vazquez, M., Colloms, S. & Sumners, D. Tangle analysis of Xer recombination reveals only three solutions, all consistent with a single three-dimensional topological pathway. *J. Mol. Biol.* **346**, 493–504 (2005).

[14] Ernst, C. & Sumners, D. W. A calculus for rational tangles: applications to DNA recombination. *Math. Proc. Cambridge Philos. Soc.* **108**, 489–515 (1990).

[15] Schlick, T. *Molecular Modeling and Simulation: An Interdisciplinary Guide.* Interdisciplinary Applied Mathematics (Springer-Verlag New York, 2010).

[16] Vologodskii, A. *Biophysics of DNA* (Cambridge University Press, Cambridge, UK, 2015).

[17] Madras, N. & Slade, G. *The Self-Avoiding Walk* (Modern Birkhäuser Classics, Cambridge, MA, 1996).

[18] Szafron, M. *Monte Carlo Simulations of Strand Passage in Unknotted Self-Avoiding Polygons.* Master's thesis, Department of Mathematics and Statistics, University of Saskatchewan (2000).

[19] Szafron, M. *Knotting statistics after a local strand passage in unknotted self-avoiding polygons in Z3.* Ph.D. thesis, Department of Mathematics and Statistics, University of Saskatchewan (2009).

[20] Orlandini, E. *Monte Carlo Study of Polymer Systems by Multiple Markov Chain Method, in Numerical Methods for Polymeric Systems*, 33–57 (Springer New York, New York, NY, 1998). URL `http://dx.doi.org/10.1007/978-1-4612-1704-6_3`.

[21] Fishman, G. *Discrete-event simulation: modeling, programming, and analysis* (Springer-Verlag, London, 2001).

[22] Scharein, R. *et al.* Bounds for the minimum step number of knots in the simple cubic lattice. *J. Phys. A : Math. Theor.* **42**, 475006 (2009).

[23] Krzywinski, M. I. *et al.* Circos: an information aesthetic for comparative genomics. *Genome Res.* **19**, 1639–1645 (2009).

[24] Ishihara, K. & Shimokawa, K. Band surgeries between knots and links with small crossing numbers. *Prog. Theor. Phys. Supplement* **191**, 245–255 (2011). arXiv:http://ptps.oxfordjournals.org/content/191/245.full.pdf+html.

[25] Ishihara, K., Shimokawa, K. & Vazquez, M. *Site-specific recombination modeled as a band surgery: applications to Xer recombination*, 387–401. Nat. Comput. (Springer, Heidelberg, Berlin, Heidelberg, 2014). URL http://dx.doi.org/10.1007/978-3-642-40193-0_18.

[26] Yoshida, M. *Applications of band surgery and signed crossing changes of knots and links to molecular biology.* Master's thesis, Department of Mathematics, Saitama University (2013).

[27] Scharein, R. G. *Interactive topological drawing.* Ph.D. thesis, Department of Computer Science, The University of British Columbia (1998). URL https://open.library.ubc.ca/cIRcle/collections/831/items/1.0051670.

[28] Brasher, R., Scharein, R. G. & Vazquez, M. New biologically motivated knot table. *Biochem Soc. Trans.* **41**, 606–611 (2013).

[29] Doll, H. & Hoste, J. A tabulation of oriented links. *Math. Comp.* **57**, 747–761 (1991).

[30] Freyd, P. *et al.* A new polynomial invariant of knots and links. *Bull. Amer. Math. Soc.* **12**, 239–246 (1985).

[31] Przytycki, J. H. & Traczyk, P. Conway algebras and skein equivalence of links. *Proc. Amer. Math. Soc.* **100**, 744–748 (1987).

[32] Cromwell, P. R. *Knots and Links* (Cambridge University Press, Cambridge, UK, 2004).
